# The Influence of Hybridization of Epoxy–Glass Laminates Modified with Metal Oxides and Graphite Particles

**DOI:** 10.3390/ma17133175

**Published:** 2024-06-28

**Authors:** Cezary Drenda, Przemysław Nosal, Kamil Badura, Patrycja Bazan

**Affiliations:** 1 Department of Machine Design and Maintenance, Faculty of Mechanical Engineering and Robotics, AGH University of Krakow, al. A. Mickiewicza 30, 30-059 Krakow, Poland; drenda@agh.edu.pl (C.D.); pnosal@agh.edu.pl (P.N.); 2 SHM System Sp. z o.o., Sp. kom. Libertów, ul Jana Pawła II 82A, 30-444 Krakow, Poland; kb@shmsystem.pl; 3 Chair of Material Engineering and Physics, Cracow University of Technology, 31-155 Krakow, Poland

**Keywords:** epoxy–glass laminate, thermal conductivity, mechanical properties, aging

## Abstract

This study examined the impact of hybridization on the mechanical properties of glass–epoxy laminates by incorporating metal oxides and graphite particles into the resin matrix. Basic mechanical tests were conducted, followed by accelerated thermal aging tests. Results showed an increase in bending strength ranging from 12% to almost 30% depending on the used additive. Static tensile tests indicated a 10% increase in strength for materials modified with flake graphite. Accelerated aging tests resulted in a 20% decrease in elastic modulus and 10% decrease in tensile strength. Additives did not improve tensile strength but increased stiffness by 30% for laminates with flake graphite. Fatigue and conductivity tests were also performed, revealing enhanced thermal conductivity and reduced impedance in materials modified with graphite flakes. The study suggests that additives can enhance the mechanical properties of glass–epoxy laminates, making them suitable for applications in automotive and aerospace industries.

## 1. Introduction

Epoxy resin laminates are widely employed in numerous sectors because of their remarkable mechanical properties and diverse application prospects. These laminates exhibit superior mechanical strength and good dynamic impact resistance, rendering them suitable materials for sophisticated applications. When subjected to impact, epoxy laminates show a different behavior, in which broken fibers and matrix debris form a locking mechanism that prevents the propagation of cracks and delamination [1]. This aspect is essential in preserving the structural stability of the components when they are subjected to impact. To enhance the mechanical characteristics of epoxy composites, the incorporation of additional materials is a potential solution. Research has shown that bamboo–epoxy laminates exhibit superior mechanical properties compared to wood–epoxy laminates [2]. Researchers have conducted extensive studies on epoxy laminates, particularly carbon–epoxy and glass–epoxy combinations. These composites exhibit exceptional strength characteristics and are crucial for maintaining the structural integrity of laminate structures [3]. Epoxy resins are widely utilized in various industries, including the electrical and electronic sectors, as encapsulation materials, laminates, and adhesives. The versatility of epoxy laminates, combined with their structural application and increasing demand, highlights their prominence in the aerospace sector, particularly in the use of carbon–epoxy composites [4]. This underscores the importance of epoxy laminates in challenging and demanding environments, such as those found in the aerospace industry.

Research on epoxy laminates that incorporate glass fiber reinforcement has shown that this type of enhancement significantly enhances the mechanical and performance characteristics of these materials. Specifically, the incorporation of glass fibers into epoxy composites has been shown to improve their tribological behavior, load-bearing capacity, and resistance to abrasion and wear [5,6]. Glass-fiber-reinforced epoxy composites have demonstrated superior mechanical properties compared to alternative reinforcements, such as basalt fabric [6]. The extensive study of glass fiber reinforcement in polymer composites has focused on examining its impact on mechanical properties under various environmental conditions [7].

Glass-fiber-reinforced epoxy composites have specialized applications in various industries, particularly in superconducting tokamak applications, where they are essential to insulate metal components [8]. The addition of glass fibers to hybrid composites enhances shear modulus and specific mechanical attributes. The arrangement of glass-fiber-reinforced epoxy laminates affects their bending properties and overall mechanical performance [9,10]. Studies have shown that the tensile strength of glass-fiber-reinforced polymer (GFRP) may decrease in alkaline/salt environments, emphasizing the importance of reducing exposure to high pH levels for the durability of GFRP. Empirical functions have been proposed to quantitatively describe the degradation of GFRP, helping engineers design durable composites [11,12]. Incorporating fibers such as Kevlar, carbon, and glass into an epoxy resin matrix improves the properties of glass epoxy laminates, enhancing tensile strength, damping response, bending characteristics, and impact resistance. The arrangement of layers in these composites is crucial for achieving the desired strength properties [13,14,15,16,17,18,19,20,21,22]. Furthermore, the addition of nano- and microparticles, such as layered silicates and metal particles, further enhances the flexural strength, thermal behavior, impact resistance, and energy absorption properties of glass epoxy laminates [23,24,25,26,27].

Epoxy laminates are indispensable in several industries due to their exceptional mechanical properties, impact resistance, and adaptability. Comprehending their behavior under diverse circumstances and improving material combinations are critical for optimizing their performance and dependability.

The primary objective of this research was to evaluate the mechanical properties and conductivity of glass–epoxy composites. In pursuit of this goal, the study utilized graphite in the form of gunpowder and flakes, as well as copper and aluminum in the form of gunpowder. The findings were compared with a reference material, which was a glass–epoxy composite. The study included fundamental tests for mechanical properties, such as static tensile and bending tests. In addition, the fabricated composites underwent an accelerated aging process to assess their resistance to environmental degradation. The study also involved accelerated fatigue tests and measurements of thermal and electrical conductivity. These materials, with their enhanced strength, low density, and heightened thermal and electrical conductivity, can be utilized in various industries because of their electrically dissipative properties.

## 2. Materials and Methods

### 2.1. Materials

The test samples were manufactured using LG206 epoxy resin and HG359 hardener (GRM Systems, Bielsko-Biała, Poland), copper oxide particles (Suzhou Canfuo Nanotechnology Co., Ltd., Suzhou, China), aluminum oxide (Suzhou Canfuo Nanotechnology Co., Ltd., Suzhou, China), and two types of graphite flake and powder (Grafity SINOGRAF, Toruń, Poland). The resin-to-hardener weight ratio was 100:34, and the reinforcement content corresponded to 10% of the weight (Table 1). The sample production process involved accurately weighing the proportions of resin and hardener on a Radwag 22 W laboratory scale (Radom, Poland), and then mixing the ingredients using a SH-II-7C series mechanical digital mixer up to 40 L, with a power of 80 W (Chemland, Stargard, Poland). Mixing was conducted at a speed of 500 rpm by gradually adding additives. To achieve a homogeneous suspension, a dispersion disk stirrer with a diameter of 5 cm was utilized. The mixing time lasted approximately 5 min after the addition of the modifiers. Following the mixing process, the mixture was placed in a vacuum chamber. During sample production, a VC3028AG vacuum chamber (VacuumChambers, Białystok, Poland) was employed to minimize porosity resulting from factors such as mixing the ingredients. The mixture was deaerated at a pressure of 10 mbar (abs).

**Figure 1:**
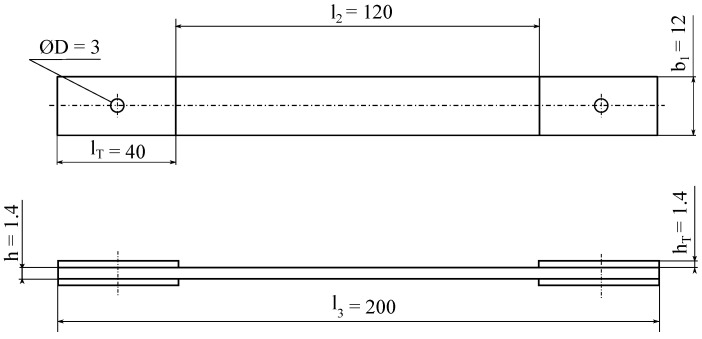
Sample geometry used in mechanical testing.

The samples were manufactured employing a manual method. The production process commenced with the placement of a fiberglass fabric with an orientation of 0/90 and a weight of 350 $\frac{g}{{\mathrm{cm}}^{2}}$ in the direction of 0° onto an acrylic sheet coated with a release agent. During the manufacturing process, a resin mixture comprising a hardener and a modifying additive was applied using a spatula, followed by the utilization of a special metal roller to compact the laminate to better cover the fabric. After saturating the fabric with the mixture, an additional layer was applied, weighing 200 $\frac{g}{{\mathrm{cm}}^{2}}$ and oriented at a 0/90 angle. This layer was placed at a 45° angle relative to the initial layer. The process was repeated until all the glass mats were laid along with the delaminating fabric. The resulting laminate configuration was [0/45/0/45/0] T. The crosslinking process was carried out at room temperature for a period of 72 h. Following crosslinking, the samples were cut to measure 200 × 200 × 1.4 mm, intended for thermal conductivity tests. The specimens for strength tests were cut using abrasive water jet technology and a water abrasive jet, with dimensions of 200 × 12 × 1.4 mm. The sample’s geometry is presented in Figure 1.

### 2.2. Methods

The primary desirable characteristics of composite materials are not only their low weight and high strength but also their ability to change based on external conditions. The density of the composites was determined using the hydrostatic method on a RADWAG WAS 22W laboratory scale (Radom, Poland).

Both static tension and static bending tests were performed. A static tensile test (compliant with the PN-EN ISO 527-1:2010 specifications) and a static bending test (according to the PN-EN ISO 178:2011 guidelines) were conducted using a Shimadzu AGS-X 10 kN testing machine (manufactured in Kyoto, Japan) at a test speed of 10 mm/min. The thermal aging process was followed by the repetition of all strength tests. The accelerated aging tests were carried out in a QUV ACCELERATED WEATHERING TESTER aging chamber (Q-LAB Corporation, Westlake, OH, USA), utilizing a research method that simulates the aging processes of materials based on temperature and humidity. The aging chamber was used for accelerated thermal aging, simulating the damage caused by sunlight and rain that can occur under atmospheric conditions over several months or years. The samples were exposed to alternating cycles of UV radiation and moisture under elevated temperature conditions to perform this test. There are various types of damage that may occur, such as alterations in color, cracks, brittleness, loss of strength, and oxidation. In order to assess the effects of aging, tests were performed according to the procedure outlined in Table 2, and this process was repeated for a period of 1000 h.

To further enhance the evaluation of the accelerated aging process, a measurement of the color change was carried out. The samples were assessed using a 3nh calorimeter (ColorCab5 ANTICORR light chamber, Gdańsk, Poland) with TL84 or 4100 K light color, which simulates typical lighting conditions found in stores, offices, supermarkets, and exhibitions. Lab color space refers to a color model that extends between opposing colors and consists of three dimensions: L, which represents brightness, and a and b, which are based on nonlinearly compressed coordinates of the CIEXYZ color space. The rectangular coordinates of a and b define the principal color axes, with red on positive a and green on negative a, and yellow on positive b and blue on negative b. This model is useful for representing intermediate shades between the primary colors of red, yellow, green, and blue. The CIE Lab color difference model was utilized to assess the color change, and the difference in color was calculated using the following formula:

(1) $$\Delta A=\sqrt{{\left(\Delta L\right)}^{2}+{\left(\Delta a\right)}^{2}+{\left(\Delta b\right)}^{2}}$$

ΔA —Euclidean distance between two points in three-dimensional space.

The observer is considered to recognize a color difference based on

0 < ΔA < 1—does not see the difference;
1 < ΔA < 2—the experienced observer will see the difference;
2 < ΔA < 3.5—the inexperienced observer can see the difference;
3.5 < ΔA < 5—the observer notices a clear difference in colors;
5 < ΔA—the observer perceives the colors as completely different.

Fatigue tests were carried out using a standard electrohydraulic servo fatigue testing machine from the Shimadzu EHF-E Series (Kyoto, Japan). The minimum stress, which remained constant throughout all load cycles, was set at 2 MPa. The maximum stress in the first cycle was set at 30% of the composite’s strength. One load block comprised 5000 cycles, after which the stress increased by 10% of the composite’s strength in subsequent load blocks. The cyclic load frequency was 10 Hz.

A thermal conductivity test was performed using an HFM 446 Lambda Eco-Line (NETZSC, Sleb, Germany) plate apparatus. This test involved placing the sample between two heat sources and monitoring the temperature of the sample at multiple locations. Through this test, it was possible to determine the amount of heat absorbed by the sample and the heat conduction ability of the material. To measure electrical conductivity, the Impedance Spectrum Analyzer IM6 device from ZAHNER-elektrik GmbH & Co (Gundelsdorf, Germany) was used. This device enabled measurements with a current frequency of up to 8 MHz and a current of up to approximately 3 A. The impedance values were read at a frequency of 5 kHz.

## 3. Results and Discussion

### 3.1. Physico-Mechanical Properties

**Figure 2:**
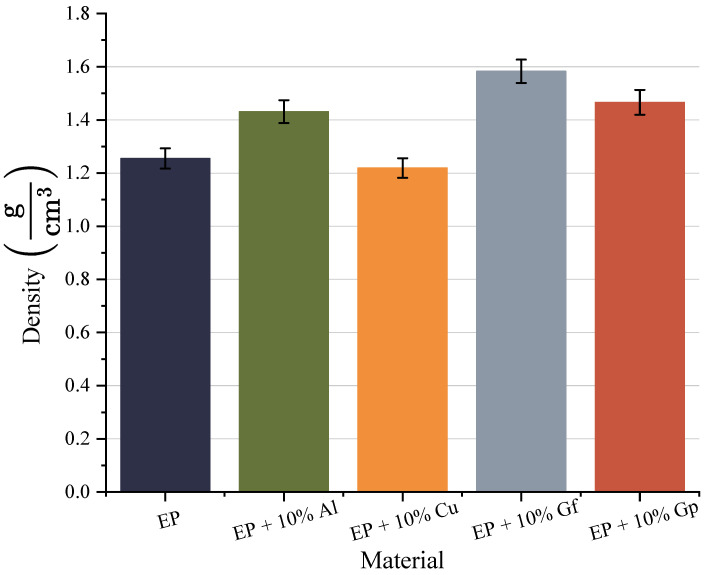
Comparison of density values of manufactured composites.

The findings of the density tests are shown in Figure 2. The data provided indicate that the composite containing graphite in flake form achieved the highest density, measuring 1.583 $\frac{g}{{\mathrm{cm}}^{3}}$, a rise of approximately 0.3 $\frac{g}{{\mathrm{cm}}^{3}}$ compared to the reference sample. In particular, the composite incorporating copper oxide exhibited the lowest density value of 1.219 $\frac{g}{{\mathrm{cm}}^{3}}$. The densities of the composites incorporating aluminum oxide and graphite in powder form were approximately 1.4 $\frac{g}{{\mathrm{cm}}^{3}}$.

**Figure 3:**
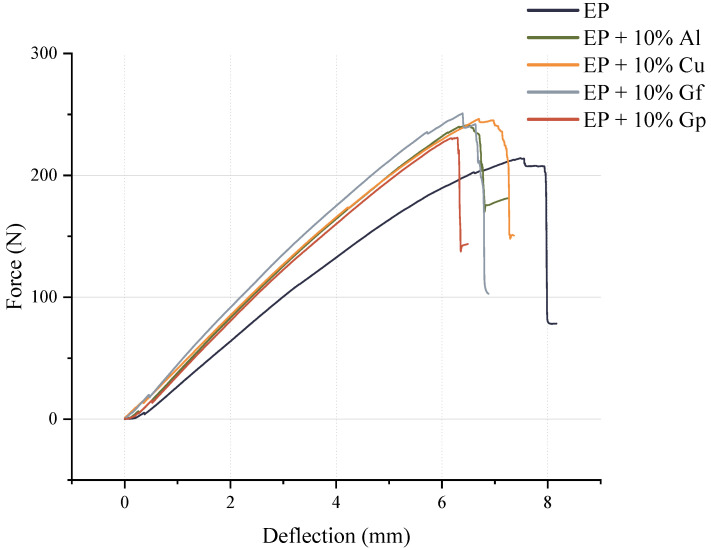
Examples of bending curves.

**Figure 4:**
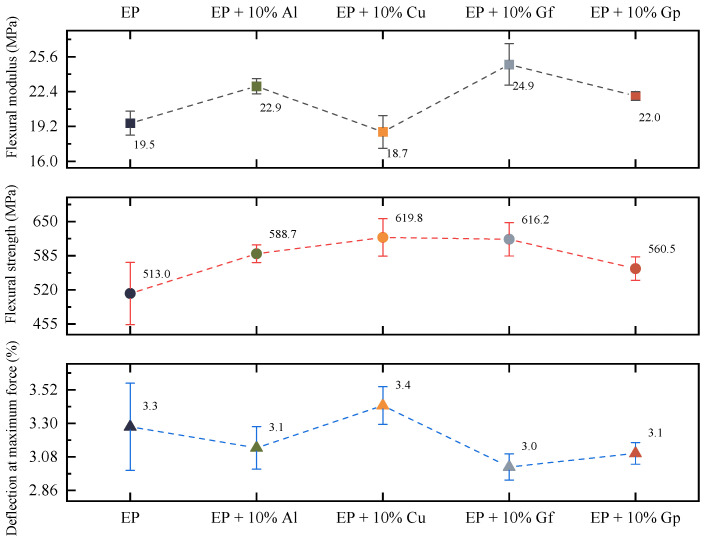
Average values of bending strength parameters.

The initial mechanical evaluation involved a static bending test, which allowed the determination of fundamental strength parameters, including flexural strength, flexural modulus, and deformation at maximum force. The results of the investigation are illustrated in Figure 3 and Figure 4. Figure 3 presents an illustration of the curves recorded during a static bending test, depicting the relationship between deflection and force. It was observed that the incorporation of additives into the laminates was observed to decrease the potential for deflection in the composites, although more force was required to destroy the materials.

Figure 4 displays the average values of the fundamental strength parameters. Based on the flexural strength diagram, it can be inferred that the laminate with the highest flexural strength was the one with copper oxide doping, but the composite incorporating a filler in the form of graphite flakes did not exhibit much variation from the highest value. The flexural strength was approximately 620 MPa; it can be observed that the reference sample without any filler had a strength lower by over 100 MPa compared to the composites with the addition of copper oxide or graphite. The highest modulus of elasticity at bending was exhibited by a laminate with graphite flakes (25 GPa) and aluminum oxide powder (23 GPa). The study by Megahed et al. [28] investigated the influence of incorporating nanometer- and micrometer-sized aluminum particles into glass-fiber-reinforced epoxy composites. The addition of aluminum significantly improved the mechanical properties of the composites, including tensile strength, flexural strength, hardness, wear, and impact strength. The most significant benefits were observed with the addition of 4% by weight of nanometer aluminum particles, which increased the tensile strength by 114%, elongation by 116%, elastic modulus by 21%, strength by 52.2%, deformation by 21.4%, and elastic modulus by 76.6%. The microscopy results confirmed the good distribution of aluminum particles in the epoxy matrix and their good adhesion to glass fibers. The addition of aluminum in micrometers also showed improvement, particularly with the addition of 4 wt% aluminum particles. The results of this study suggest that the addition of aluminum particles can greatly improve the mechanical properties of glass-fiber-reinforced epoxy composites [29]. The incorporation of copper oxide powder resulted in a reduction in the stiffness of the laminates, even when compared to the reference sample, from 19.5 GPa to 18.7 GPa. All materials demonstrated similar deformability coefficients at maximum force, but the materials with the highest deformation were the copper-oxide-doped laminate (3.4%) and the undoped laminate (3.3%). The remaining composites showed deformation at failure within 3%. Incorporation of microparticles in laminates is crucial to enhance several mechanical characteristics, such as damping, buckling load, bending stiffness, and flexural strength [30,31,32,33,34].

**Figure 5:**
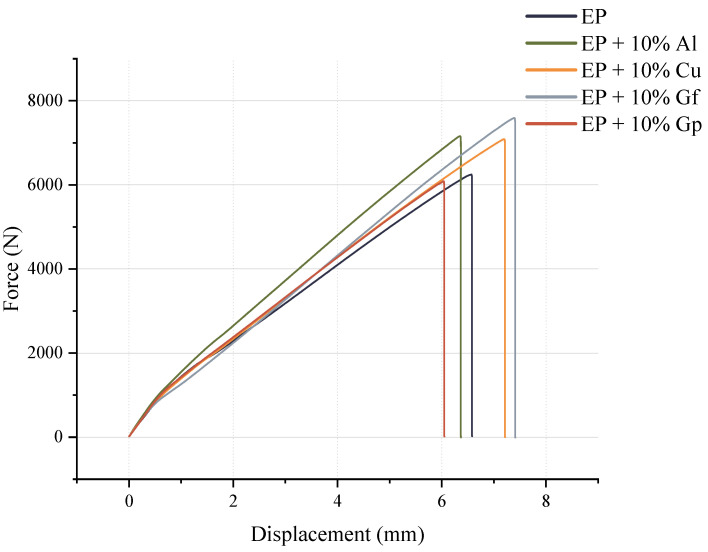
Examples of tensile curves.

**Figure 6:**
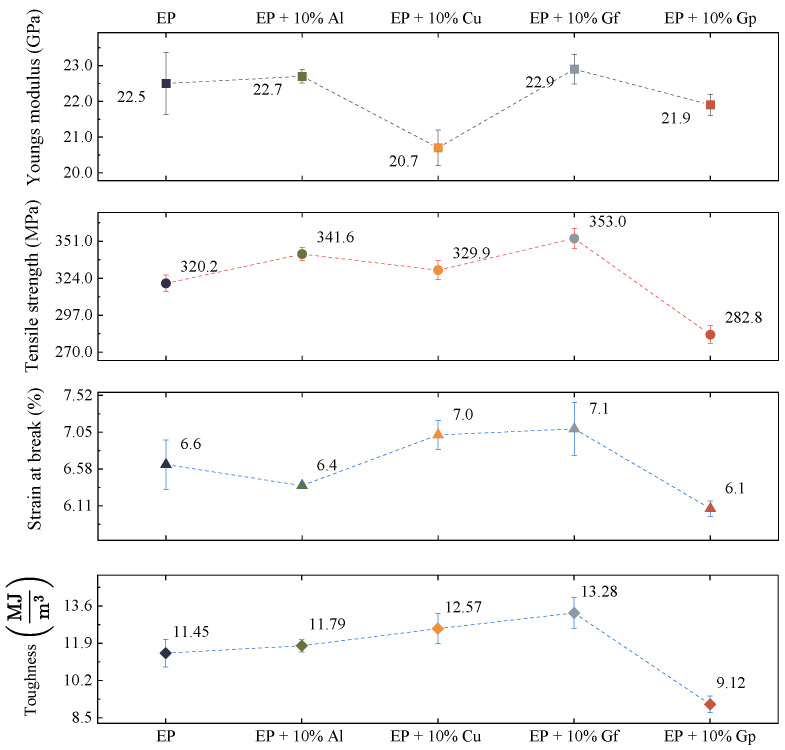
Average values of tensile test parameters.

Figure 5 and Figure 6 illustrate the results of the static tensile test. Figure 5 exhibits instances of tensile curves captured throughout the tests, while Figure 6 presents the average values of tensile strength, Young’s modulus, strain-breaking, and toughness values.

The examined data reveal that composites reinforced with aluminum powder and graphite flakes possess higher tensile strength compared to the reference sample, exhibiting an increase of 20–30 MPa. The laminate enhanced with copper oxide also demonstrates superior tensile strength. However, the incorporation of graphite powder results in a decrease in tensile strength of approximately 10%. The graphite-doped laminate in the form of flakes exhibits the highest modulus of elasticity, reaching nearly 23 GPa, a slight increase compared to the undoped sample at 0.4 GPa. The stiffness of the laminate filled with graphite in the form of gunpowder is the lowest, with a Young’s modulus of 21.9 GPa. The laminate doped with graphite in flakes and copper oxide powder demonstrates similar deformation values, with the former achieving a value of 7.1% and the latter a value of 6.1%. The test outcomes indicate possible defects emerging during the production of samples incorporating graphite in the form of powder, which could lead to accelerated delamination. Moreover, the composition and density of the individual fillers must be considered. The fillers were added to the laminate by weight, resulting in varying volume fractions for the components 3.41% for aluminum oxide, 2.16% for copper oxide, 7.19% for graphite flakes, and 5.81% for graphite powder. Notably, there is a greater volume of graphite particles in the composite compared to, for instance, copper particles. The crucial aspect in this context involves the volume fraction and particle size, together with their form and chemical makeup. As demonstrated by the test presented results, incorporating particles into laminates can yield differing effects on tensile properties, with certain particles enhancing tensile strength while others may exhibit a limited impact. The arrangement of laminates and fiber hybridization also play a significant role in determining the tensile behavior of composite materials. Studies in the literature indicate that graphite particles hold significant importance in enhancing the mechanical properties of composites. Several investigations have emphasized the positive influence of graphite particles on the mechanical properties of composites. The incorporation of graphite particles has been demonstrated to improve the strength of composites [35], improve ductility, sliding wear resistance, and ultimate tensile strength [36,37]. Uppin et al. have highlighted the significant impact of graphite oxide on not only the mechanical properties and structure, but also on the glass transition temperature, Tg. The findings suggested a decrease in the cross-linking density and storage modulus of the composite with an increase in the additive concentration. Additionally, it was observed that the dissipation of energy and wear rate of the composite decreased as the concentration of graphite oxide increased [29]. Graphite reinforcement has also been demonstrated to diminish frictional forces in dry conditions, and it also decreases internal frictional forces occurring between the composite components. The mechanical properties of composites are impacted by various factors, including particle size, shape, load, and microstructure of the matrix. Research has indicated that smaller graphite particles result in greater composite strength, which is linked to the interlocking of polymer chains [35], while the size, shape, and fraction of graphite particles, as well as the microstructure of the matrix, play a significant role in determining the mechanical properties [38]. Graphite’s shape likely influences its strength properties in materials. Elongated flakes of graphite are more compatible with the matrix material and glass fabric, whereas powdered graphite may act as additional notches and cause defects and discontinuities in the material. Cho et al. explored the influence of matrix reinforcement with graphite nanoparticles on the mechanical properties of fiber–carbon/epoxy composites. The study revealed that the addition of 3% and 5% of graphite nanoparticles improved the compressive strength of the composite material. The three-component epoxy system was used as the matrix material. The graphite particles had a diameter ranging from 0.5 to 5 μm and a thickness of 100–200 nm. The research findings indicate that reinforcing the matrix with nanoparticles can enhance the properties of composites, but the extent of improvement depends on the processing method, type, surface condition, concentration, and dispersion of the nanoparticles [39]. The influence of particles additives on laminate tensile properties was the focus of other research. Bozkurt et al., examined the effect of various particles, such as clay, silica nanoparticles, and ${\mathrm{CaCO}}_{3}$ derived from spent eggshells, on the tensile behavior of composite laminates. Studies on glass-fiber-reinforced layered clay/epoxy nanocomposites revealed that the addition of clay had little effect on tensile properties [24]. On the contrary, the addition of calcium carbonate particles to the basalt fiber/epoxide resin composites resulted in a noticeable enhancement of tensile and flexural properties, potentially attributed to the chemical reactions that occurred between the material components [40]. Halder et al. conducted a study to examine the impact of modified ${\mathrm{ZrO}}_{2}$ nanoparticles on the mechanical properties of glass-poxy laminates. The results of microscopic examinations strongly suggested that the use of silanized ${\mathrm{ZrO}}_{2}$ nanoparticles prevented particle aggregation and increased their size. The incorporation of silanized ${\mathrm{ZrO}}_{2}$ nanoparticles led to a significant improvement in the mechanical properties of the glass fiber hybrid composite, resulting in an increase in tensile strength, stiffness, and strength of approximately 27%, 62%, and 110% compared to GFRP. Additionally, the use of ${\mathrm{ZrO}}_{2}$ nanoparticles resulted in reduced interfacial delamination, which suggests improved interfacial bonding [41].

Tensile toughness expresses the energy needed to fracture the material during stretching. This energy can be obtained by the integration of the stress–strain curve [42].

(2) $$\tau =\int_{0}^{\varepsilon_{f}}\sigma d\varepsilon$$

where $\varepsilon_{f}$ is a strain at the failure. The results for considered composites are presented in Figure 6. The toughness values calculated for the prepared composites with additives are higher compared to the base laminate. Only the addition of graphite in the form of particles shows a much lower value of this parameter. This is a consequence of the lower elongation at break and the low strength. In turn, the addition of graphite in the form of flakes has the highest toughness value, which is 13.28 MJ/m^3^ and is 1.83 MJ/m^3^ higher than the base laminate. Although the volume fraction of copper oxide is lower than in the case of aluminum oxide, its greater impact on the toughness of the composite is visible. The addition of graphene in the form of flakes is characterized by the highest toughness value, but its volume fraction compared to copper oxide is more than three times higher.

Incorporation of particles into a glass–epoxy laminate alters its mechanical properties for several reasons. Nanoparticles or microfibers, for example, can enhance the fracture resistance, hardness, and tensile strength of the epoxy matrix. By adding particles to epoxy laminates, the strength of the composite is increased, resulting in improved energy dissipation and greater resistance to mechanical damage. This fortification is attributable to mechanisms such as bridging particles, pinning or bending cracks, and altering the crack path [43]. The addition of inorganic materials such as graphite to epoxy nanocomposites enhances the mechanical properties of the polymer, with the optimal loading amount being up to 1 wt%. This results in improved strength and mechanical performance of the material [44]. In addition, the incorporation of particle guidance has the potential to strengthen the bond between the glass fiber layers and the epoxy matrix. This leads to more efficient stress transfer between composite components, ultimately resulting in increased structural strength. Swikker et al. have explored the influence of multi-walled carbon nanotubes (MWCNTs) on the mechanical properties of glass/carbon hybrid composites. Their research demonstrates that the addition of grafted MWCNT in glass fabrics is associated with improved mechanical properties and enhanced reinforcement. This suggests that the inclusion of particles such as graphite in glass epoxy laminates could also result in heightened mechanical properties and improved adhesion [45]. Mishra et al. conducted a study to evaluate the impact of graphene addition on the mechanical properties of glass/epoxy composites. The findings revealed that the incorporation of graphene led to a remarkable improvement in the tensile, flexural, and impact strength of the composite. The optimal results were achieved with a 2% by weight addition of graphene. The addition of graphene also resulted in better plasticity in comparison to the non-graphene laminates. However, it is important to note that uneven dispersion of graphene can adversely affect the mechanical properties of the composite. To achieve further improvements, it is suggested that the production process be optimized and the dispersion of graphene be enhanced [46]. Incorporation of particles within the laminate has an impact on the distribution of stress within the material. These particles can serve as centers for stress dissipation, which decreases the stress concentration and the likelihood of local cracking. Additionally, certain particles can enhance the material’s capacity to dampen vibrations, thereby improving its dynamic properties. This enhanced vibration damping is particularly advantageous in applications where the material is subjected to recurring loads [47,48,49].

**Figure 7:**
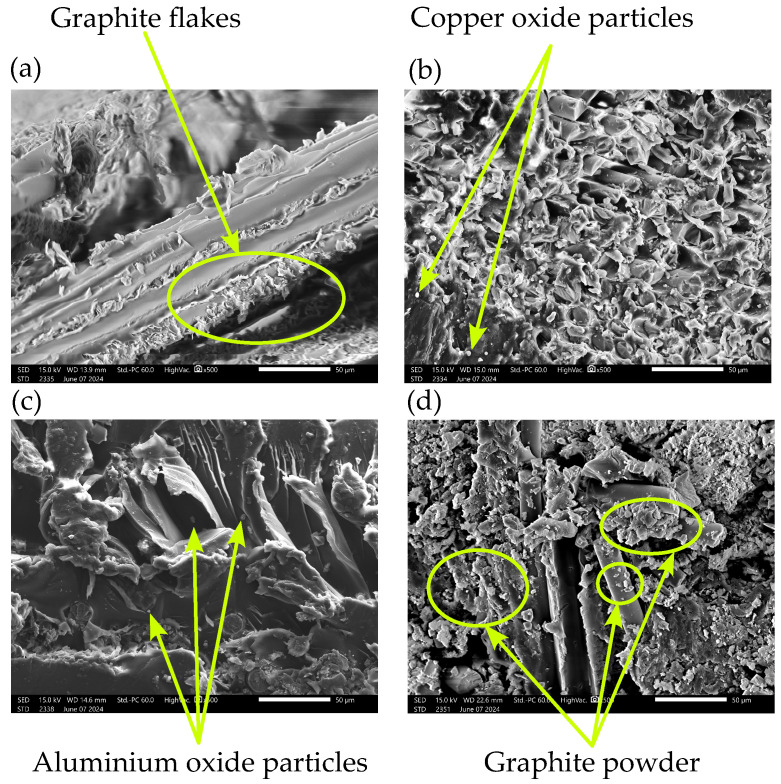
SEM microstructure: (**a**) EP + 10% Gf; (**b**) EP + 10% Cu; (**c**) EP + 10% Al; (**d**) EP + 10% GP.

The microstructure of glass–epoxy laminates that contain graphite particles is characterized by uniformly spaced glass fibers that are embedded within an epoxy matrix, which has evenly distributed graphite particles (Figure 7). The epoxy matrix envelops and binds the glass fibers, ensuring the structural stability of the composite material. The epoxy resin fills the gaps between the graphite particles and fibers, ensuring the cohesion of the material. However, graphite particle clusters were observed in the composite when graphite powder was used. Graphite particles act as bridges between glass fibers, which can cause interactions with the matrix, affecting the mechanical and electrical properties of the laminate. The presence of single pores, microcracks, and other defects resulting from the production process was also observed in the microstructure, which can impact the strength and other mechanical properties of the laminate. For composites modified with aluminum oxide and copper oxide, the dispersion of the particles was found to be heterogeneous and their number was significantly smaller, which is related to their lower volume fraction. Despite their greater stiffness compared to graphite particles, this modification appears to be insufficient for effective operation in the composite.

### 3.2. Effects of Accelerated Aging

**Figure 8:**
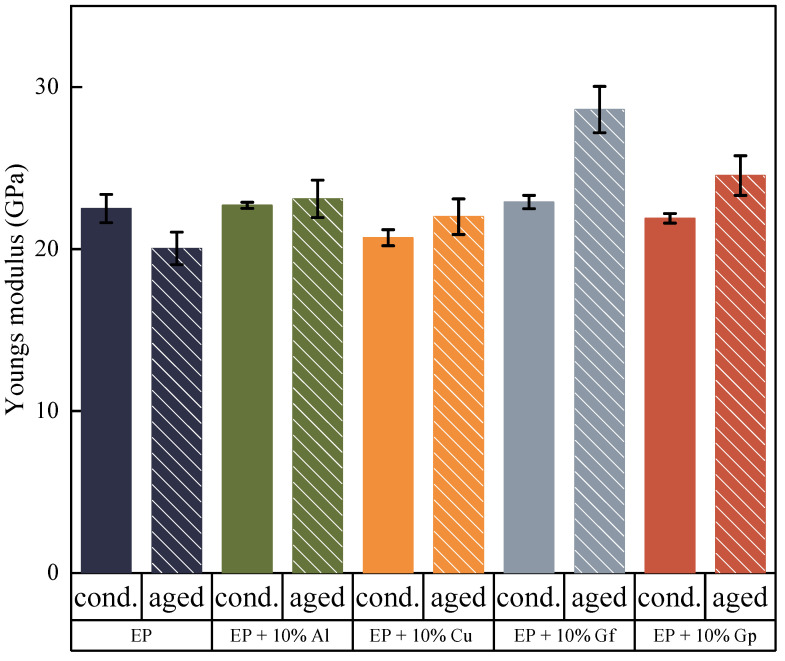
Average values of Young’s modulus.

**Figure 9:**
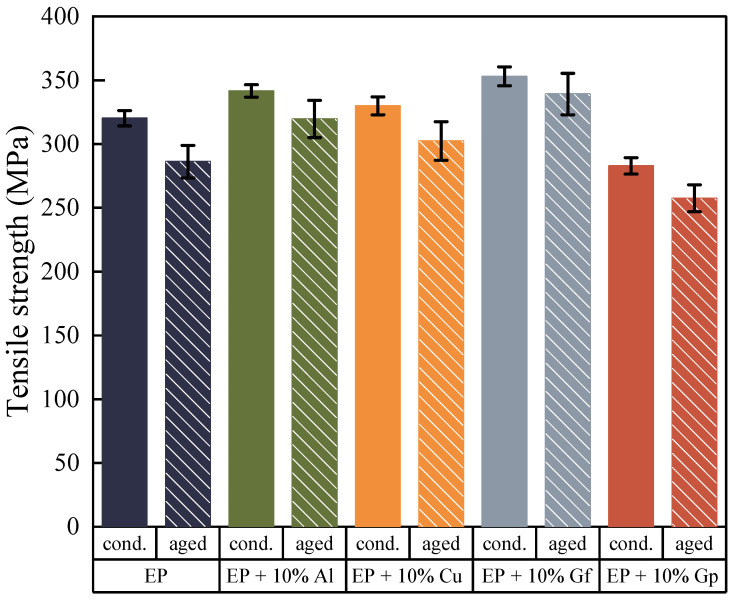
Average values of tensile strength before and after accelerated aging.

**Figure 10:**
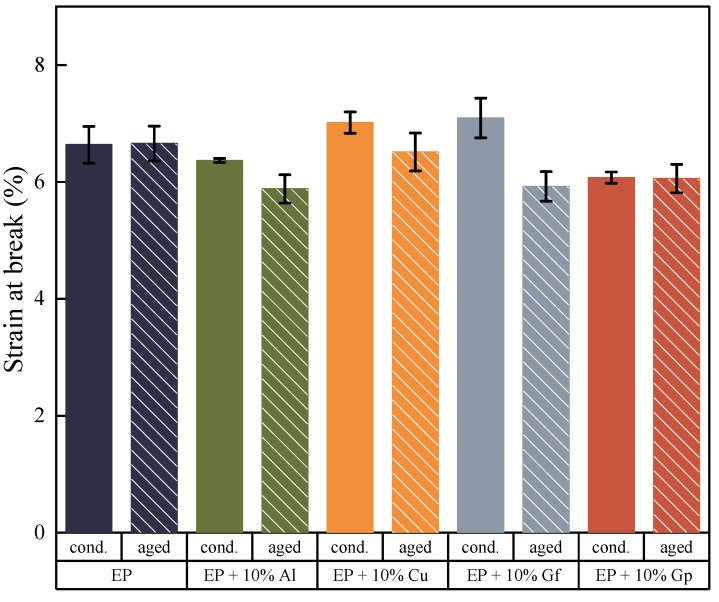
Strain at break before and after accelerated aging.

**Figure 11:**
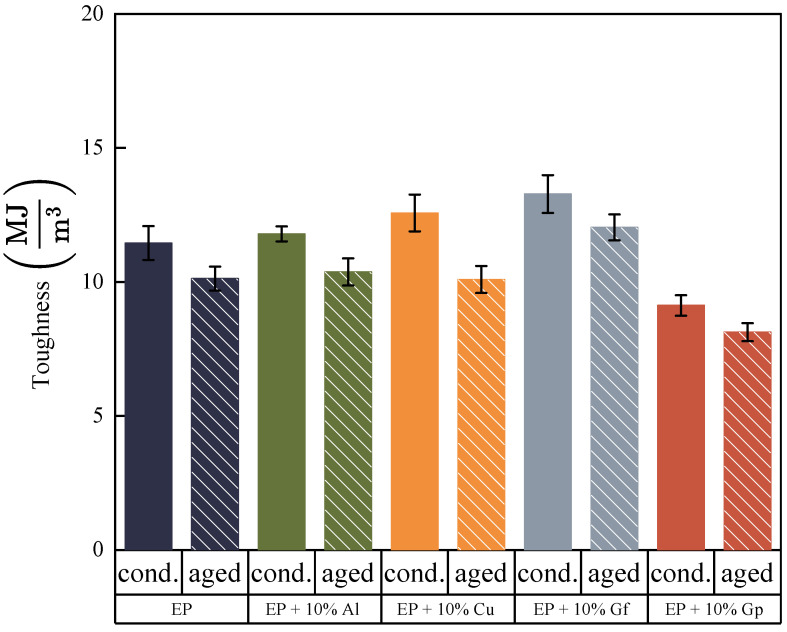
Average values of toughness before and after accelerated aging.

**Figure 12:**
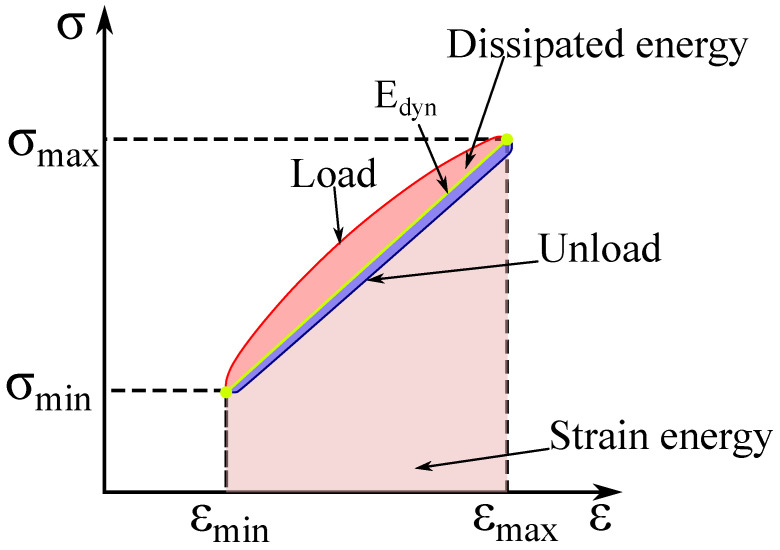
A schematic depiction of hysteresis loop with marked area related to dissipated energy.

**Figure 13:**
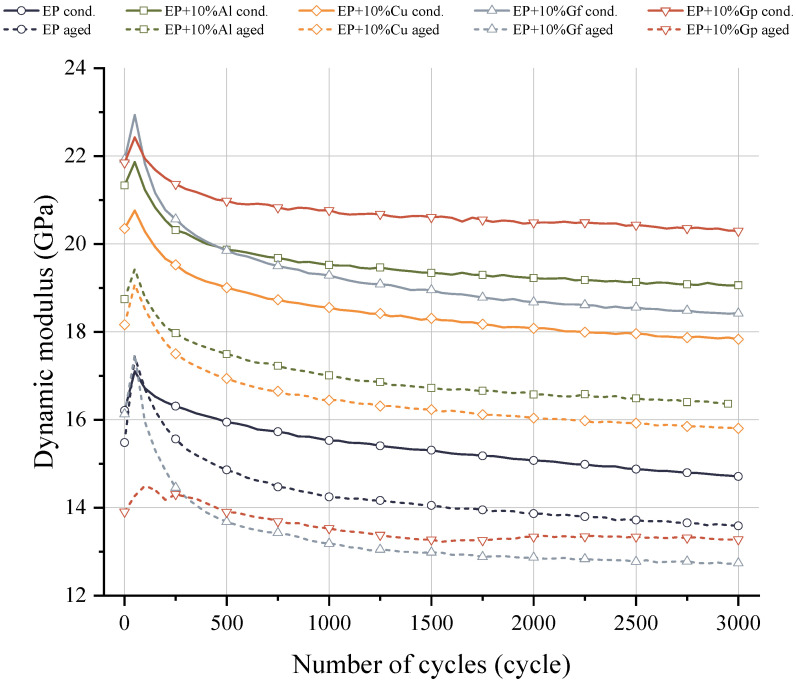
Comparison of the dynamic modulus of the composites before and after the accelerated aging.

**Figure 14:**
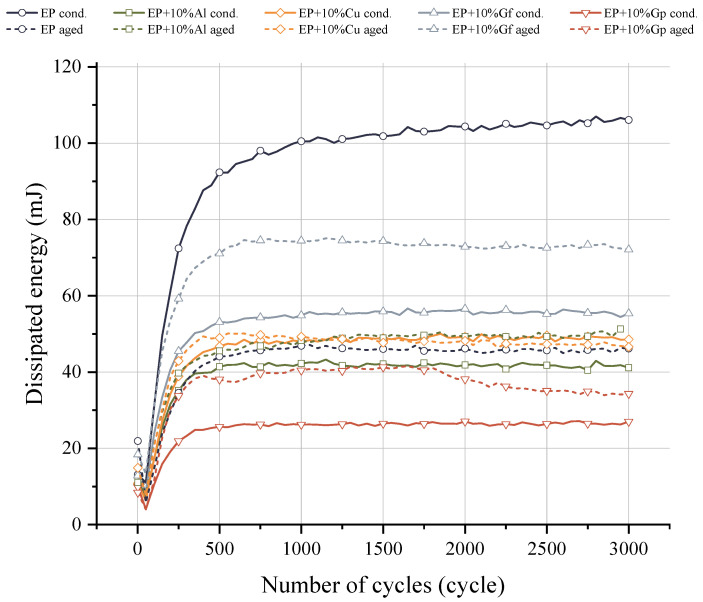
Comparison of the energy dissipation during cycling loading before and after the accelerated aging.

The study involved subjecting the fabricated materials to accelerated thermal aging by exposure to UV radiation, temperature, and humidity for a period of 1000 h. This process simulated the complex ways in which external factors can influence polymer composites. To assess the impact of these factors on the mechanical properties of the materials, the study included re-testing of the materials. The findings of the study are presented in Figure 8, Figure 9, Figure 10, Figure 11, Figure 12, Figure 13 and Figure 14. The purpose of this research was to investigate the effects of external factors on the properties of materials.

The aging process of epoxy resin involves intricate interactions that can result in changes in physical, mechanical, thermal, and chemical properties. It is imperative to comprehend these aging mechanisms to accurately predict the long-term behavior and durability of epoxy resins and their composites in numerous applications [50]. Various forms of aging are identified, emphasizing physical, hygrothermic, and thermal aging, as well as chemical reactions that impact the characteristics of epoxy resins and their composites. According to Wang et al. [51], hygrothermal aging and salt spray primarily influence the structure of the outer layer of the composite, weakening the bond between epoxy resin and outer fibers and reducing the impact strength. In addition, aging of epoxy resins can result in alterations to their mechanical, thermal, and chemical properties. According to Wang et al. [50], an extended service life can lead to several negative consequences, including yellowing, diminished gloss, cracks, and a decrease in mechanical and insulating properties.

The outcomes of the research examined vary based on the additives used. However, it can be observed that the incorporation of the additives diminishes the influence of accelerated thermal aging on Young’s modulus in relation to the glass–epoxy laminate without modifiers (Figure 8). Additionally, it was noticed that there was an increase in the modulus of elasticity after the aging process, particularly for materials modified with graphite, both in the form of flakes and particles.

The addition of copper oxide and aluminum oxide particles led to an improvement in the stability of the tested materials, both in terms of tensile strength (Figure 9), with a lower decrease compared to the reference samples, and an increase in Young’s modulus. The impact of accelerated thermal aging time on break deformation decreased for all materials (Figure 10); though, in this case, the presence of copper particles also mitigated the negative effects of the external environment on the materials tested.

The integration of copper oxide (CuO) and aluminum oxide (${\mathrm{Al}}_{2}O_{3}$) into composite materials enhances their performance and stability through different mechanisms. The presence of copper oxide can initiate exothermic reactions both on the surface and within the material, leading to cross-linking of the resin and positively impacting mechanical properties [52]. On the one hand, alumina has been demonstrated to enhance the flexural strength and water resistance of composites, which in turn improves their mechanical properties and overall durability [50,53]. Research indicates that the incorporation of copper oxide and aluminum oxide in composite materials plays a pivotal role in the development of stable architectures and protective barriers [54]. In conclusion, the incorporation of copper oxide and alumina into composite materials enhances their mechanical properties, thermal stability, corrosion resistance, and the development of protective layers. These oxides possess distinctive qualities that profoundly influence the overall characteristics and practical applications of composite materials across diverse industries.

Figure 11 shows the tensile toughness of the composites before and after aging. A decrease in the parameter value is observed for each of the composites, where the composite with the addition of copper shows the greatest degradation. However, the value obtained is similar to the reference EP composite and the composite with the addition of aluminum and is approximately 10 MJ/m^3^. The composite with the addition of Gf shows the highest toughness value after aging, similar to the analysis of unaged samples. However, the addition of graphene in the form of a powder significantly worsens the toughness value.

The review of the literature on the aging process suggests that several factors operate simultaneously. For example, elevated temperatures can promote beneficial secondary crystallization and chemical breakdown of the chain, leading to negative consequences. Physical aging is typically caused by the migration of volatile components, the absorption and desorption of water, or swelling. Chemical changes in structure are often the result of an increase in temperature in combination with oxygen or other factors, such as radiation. Elevated temperatures can initiate crosslinking, improving the thermal, mechanical, and physical properties of the material [4,55,56]. The presented results seem to indicate that the modification of humidity and temperature cycles led to an increase in strength after the accelerated thermal aging process; however, it is crucial to consider the impact of additional factors, such as particle size and shape.

The materials underwent colorimetric testing both before and following the aging process. The outcomes of these tests are summarized in Table 3. The presented results of the research showed that the epoxy–glass laminate was the most susceptible to accelerated thermal aging. The differences in color before and after the process were significant, and the colors were perceived as completely different. Materials modified with graphite and copper oxide also changed color after the aging process, but these changes were much less noticeable. The only material with which no color changes were observed was the material modified with aluminum oxide.

### 3.3. Accelerated Fatigue Testing

In the case of tests carried out with cyclic mechanical loading of the material, there is an effect of energy dissipation, which to some extent takes the form of thermal energy, which leads to the heating of the object in question, while the remaining part of the energy is dissipated by the elastoplast. The consequence of this heating is a decrease in the mechanical strength of the material as a result of structural changes. The stored thermal energy is accumulated, which consequently causes the material to soften and the polymer bonds to crack.

Figure 12 shows a diagram illustrating the hysteresis loop with the marked area corresponding to the energy that is dissipated. During fatigue tests, the stiffness modulus degrades; therefore, the dynamic modulus $E_{\mathrm{dyn}}$ was determined as the slope of the line between the limit hysteresis points, and its value was calculated from the ratio

(3) $$E_{\mathrm{dyn}}=\frac{\sigma_{\max}-\sigma_{\min}}{\varepsilon_{\max}-\varepsilon_{\mathrm{minm}}}$$

this module also takes into account viscous phenomena that occur in polymers. Additionally, the value of the dissipation energy was estimated, which corresponds to the area of the hysteresis loop (Figure 12), which is a measure of the energy loss in each cycle. This loss is related to the heating of the material and the structural reconstruction. The obtained results of the dynamic modulus and energy were used to characterize the structural state of the composites tested that were subjected to cyclic loading tests.

All the analyzed composites show an initial intensive increase in the dynamic stiffness modulus, in the range of up to 100 load cycles (Figure 13). The highest recorded stiffness value before the aging stage is recorded for the composite with the addition of graphite in the form of flakes, 22.93 GPa, while the lowest value is for the laminate without the addition of 17.1 GPa. The conduct of aging tests resulted in a degradation of the stiffness parameters, with the lowest environmental impact recorded for the EP base composite, where a slight increase in the maximum $E_{\mathrm{dyn}}$ value of 0.33 GPa was recorded. In turn, the composite with the addition of graphite particles is characterized by the greatest degradation, where the difference in maximum values is 7.92 GPa. With subsequent load cycles, gradual degradation of the dynamic stiffness modulus is noticeable, with the composite with the addition of Gf characterized by the most intense decrease in stiffness. For this composite, in the case of aged samples, the stiffness value in the last loading cycle was equal to 12.73 GPa. This value is 3.63 GPa lower compared to the composite aged with the addition of Al. Subsequent load cycles cause degradation of the stiffness modulus, which is related to the effect of the evolution of material damage. This damage may be related to the formation and development of microvoids in the composite matrix, which in turn is largely related to the loss of cohesion between the additive particles and the matrix material. Another damage mechanism may be related to the formation of cracks in the glass mat fibers.

Figure 14 shows the energy dissipated during the cyclic loading of individual composites. A characteristic feature is a decrease in energy in the initial 50 load cycles and then its increase up to the saturation level, at which no further increase in energy is noted. The EP base composite has the highest energy dissipation capacity, with an energy value of 108 mJ. However, from the group of composites containing additives, there is a composite containing graphite in the form of flakes after aging. Its dissipation energy in the stabilized stage is 72.11 mJ and is 33.95 mJ lower than that of the EP composite. However, the Gf version before aging is also characterized by a high level of energy dissipation compared to the other additives, where the energy value reaches 55.34 mJ. The lowest dissipation energy value was recorded for the Gp composite before aging. The remaining composites show a similar level of energy dissipation, with the composite with the addition of graphene powder after aging showing a reduction in energy values after 1800 cycles. An increase in the number of cycles and/or the stress ratio causes an increase in the amount of mechanical energy that is dissipated, which is explained by increased friction in the unbounded zones as a result of the increase in damage [57]. After 750 cycles, composites show stabilization of energy dissipation, with no significant changes despite repeated deformation cycles. This indicates a high level of durability and reliability in maintaining their mechanical properties. This mechanism was described by Porbębska et al. [58], who provide a robust theoretical framework for analyzing energy dissipation and mechanical behavior in thermoplastic composites, paving the way for further investigations into other composite systems and the development of innovative solutions to address cyclic weakening.

### 3.4. Thermal and Electrical Conductivity

**Figure 15:**
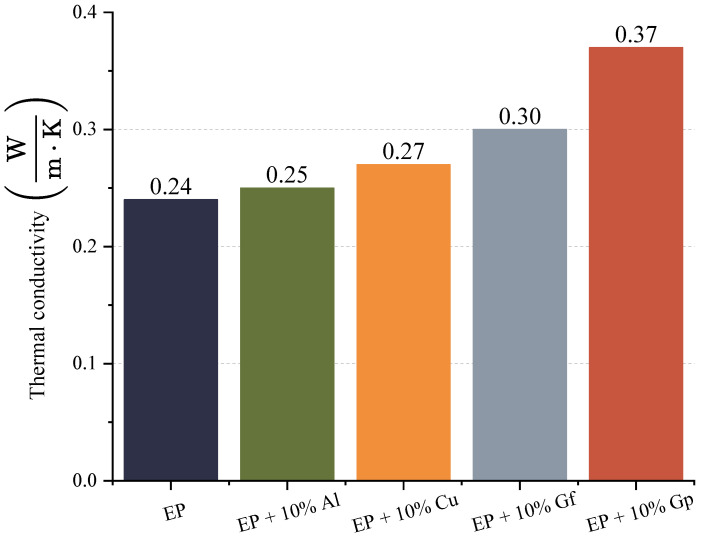
Thermal conductivity.

**Figure 16:**
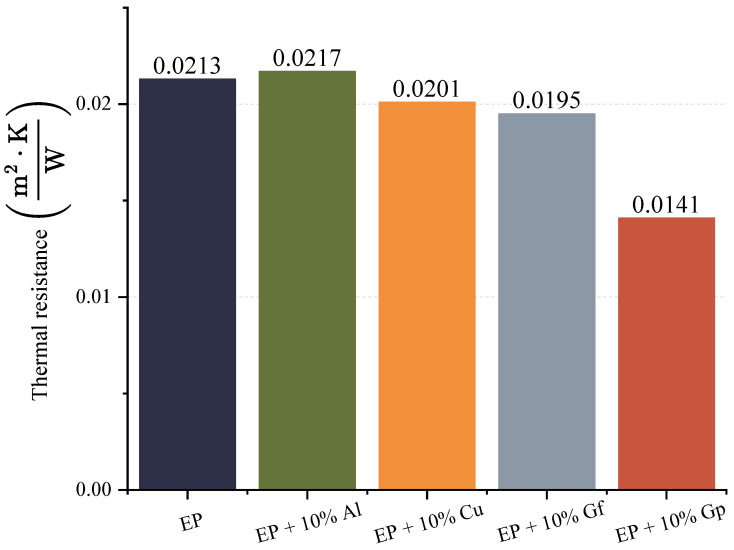
Thermal resistance.

The objective of incorporating graphite, aluminum oxide powder, and copper oxide as admixtures was to enhance the thermal and electrical conductivity of the resulting laminates. The outcomes of the thermal conductivity tests are depicted in Figure 15 and Figure 16.

The greatest thermal conductivity was observed in the material incorporated with graphite in powder form, which exhibits a substantial variation compared to the sample without a filler (Figure 15). The test outcomes indicate that the percolation threshold has been surpassed, resulting in an increase in the thermal conductivity. The percolation limit for an epoxy–graphite laminate determines the threshold at which the material becomes conductive. Ramanujam et al. found that powder composites exhibited a percolation threshold at 3 wt%. graphite, which highlights the role of graphite concentration in the electrical conductivity of the laminate [59]. According to Kranauskaitė et al., the lowest percolation threshold occurs in exfoliated graphite (EG) composites, suggesting that the addition of EG lowers the percolation limit, improving conductivity [60]. The dissimilarities in conductivity between graphite-modified laminates are primarily influenced by the geometry of the flakes. According to Maletić’s research, the percolation threshold for randomly dispersed conductive graphite flakes in an epoxy matrix is inversely proportional to the aspect ratio of the flakes. Therefore, the ratio of the lateral dimension to the thickness of the flakes plays a critical role in determining the conductivity of the material [61]. Throckmorton and Palmese noted that the percolation threshold for graphite flakes typically ranges from 0.5 to 1% by volume [62].

**Figure 17:**
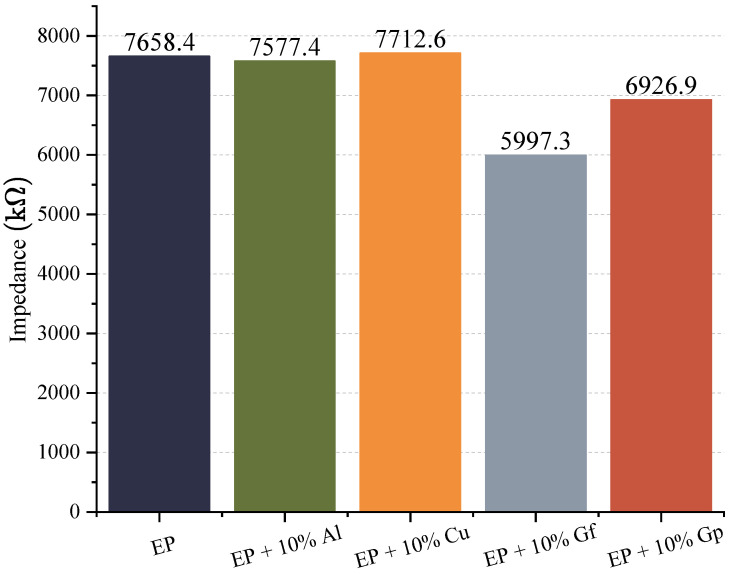
Impedance.

**Figure 18:**
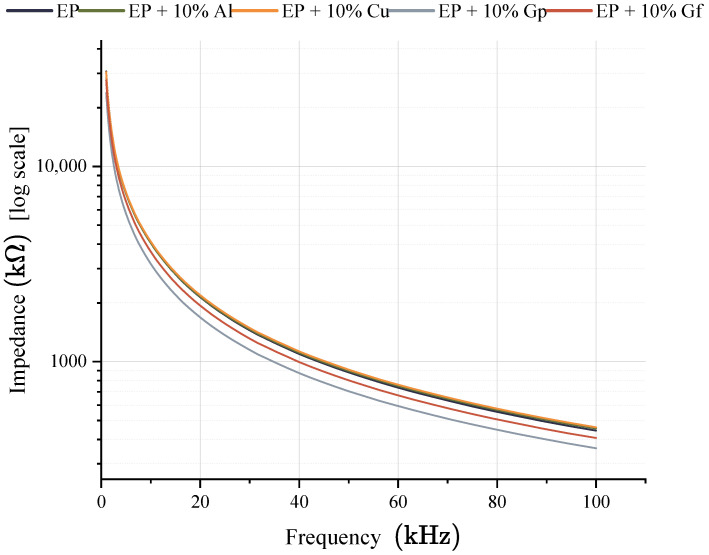
Impedance vs. frequency.

In the resistance diagram (Figure 16), it is evident that the lowest resistance required for heat transfer belongs to the laminate containing graphite in the form of powder, and the only instance where the resistance is higher than the reference sample is a laminate doped with copper oxide powder, and this difference is minor. During the research, electrical conductivity was evaluated by measuring electrical impedance. The data obtained from this process are represented in Figure 17 and Figure 18.

The functioning of the tested samples can be understood as analogous to the resistive mechanism observed in resistors, with the impedance value reflecting the opposition to alternating current displayed by the sample. The incorporation of aluminum led to a slight reduction in impedance value compared to the base material, amounting to approximately 1%. In contrast, the addition of copper particles resulted in a slight increase in impedance, approximately 0.7%, which may be attributed to the heterogeneity of the material, given that copper is a highly conductive material. The most effective additive in improving electrical conductivity turned out to be graphite, either in the form of powder or flakes with is related to the volume amount of additive in relation to materials modified with aluminum or copper oxide. The former caused a more significant decrease in impedance, approximately 20%, while the latter resulted in a smaller increase in conductivity, around 10%. The results of the tests on samples containing graphite suggest that the dispersion of particles may be more even in the case of gunpowder, while the arrangement and shape of the flakes likely play a role in influencing conductivity in samples with flake graphite. Senis et al. reported similar results. They investigated the impact of graphite oxide on the conductivity of epoxy–carbon laminates and found that adding graphite oxide in small amounts (less than 1.26% vol.) slightly increased the conductivity. However, increasing the filler content above 2.52% vol. resulted in a nonlinear increase in electrical conductivity, reaching a maximum value of approximately 0.7 $\frac{S}{\mathrm{cm}}$ at 6.3% vol. A similar increase was observed for thermal conductivity after exceeding the content of 2.52% vol., but it was linear [63].

A formal analysis of the thermal conductivity of epoxy laminates containing organic and inorganic microparticles was conducted. Chen et al. demonstrated that the thermal conductivity of epoxy/BN-PVDF composites increased to 1227 $\frac{W}{m\cdot K}$ at 21 wt% [64]. Lee et al. achieved a particularly high thermal conductivity of 3.397 $\frac{W}{m\cdot K}$ in composites by using a high content of aluminum nitride in the epoxy resin [65]. Chung et al. showed that epoxy resin composites filled with h-BN particles achieve thermal conductivity of 1.0527 $\frac{W}{m\cdot K}$ or 0.977 $\frac{W}{m\cdot K}$, depending on the surface treatment of the particles [66]. Wang et al. investigated sandwich-type epoxy–oxide composites in which the outer layers increased thermal conductivity while the middle layer maintained high puncture strength [67]. The research of Wu et al. examined the combined effect of irregular alumina and round plates of boron nitride particles on the thermal conductivity of epoxy composites. The study resulted in a significant increase in thermal conductivity of over 700%, showcasing the potential for improving the thermal conductivity of epoxy laminates by incorporating specific metal particles such as aluminum nitride and boron nitride [68]. Mahanta et al. conducted a study on the thermal conductivity of epoxy composites that included graphite and graphene fillers. The study demonstrated the effectiveness of using a hybrid graphite-graphene filler system to achieve a thermal conductivity of 42.4 ± 4.87 $\frac{W}{m\cdot K}$, which is significantly higher than that of unmodified epoxy resin. This increase in thermal conductivity was observed in a sample containing 30% by weight of graphite and 5% wt. graphene [69]. The research of Suherman et al. also investigated the electrical conductivity of epoxy laminates with graphite and showed that they could achieve high electrical conductivity of up to 65 $\frac{S}{\mathrm{cm}}$ [70]. Toselli et al. highlighted the improvement of mechanical properties and electrical conductivity in graphene-epoxy composites by functionalizing graphite nanoparticles with an epoxy monomer [71]. Kaushik et al. evaluated the mechanical and electrical properties of epoxy–graphite composites and emphasized the importance of particulate fractions in determining these properties [72]. Furthermore, the work of Wang et al. demonstrated the role of graphitization and microstructures in improving the electrical conductivity of carbon laminates, which may be relevant when considering the conductivity of graphite in epoxy composites [73].

Collectively, these results emphasize the impact of variables such as the type of filler, functionalization techniques, and composite structure on the electrical conductivity of graphite-reinforced epoxy laminates. Comprehending the electrical behavior of graphite-infused epoxy composites is essential for numerous applications, including aerospace, automotive, and electronic devices. By adjusting the formulation and processing parameters based on the findings from these investigations, it is feasible to customize the electrical conductivity of graphite epoxy laminates to meet specific requirements, ultimately ensuring optimal performance in various processes and applications.

Composite materials reinforced with metal oxide particles exhibit excellent electrical and thermal conductivity, thanks to a phenomenon known as percolation. To become a conductor, a composite must form continuous conductive paths. The formation of a conductive network enables the free flow of electrons through interconnected paths, resulting in increased thermal and electrical conductivity. The percolation threshold represents the minimum concentration of reinforcing particles required to create conductive bundles. If this value is not reached, the particles are randomly distributed within the material, causing lattice interference and hindering electron flow. This phenomenon is essential in the development of particle-reinforced composites with conductive properties, as it allows for the selection of appropriate particle concentrations [74]. In a study conducted by Misiura and colleagues, the mechanical properties, as well as the electrical and thermal conductivity, of copper–nickel-modified epoxy composites were investigated. The results revealed that the electrical conductivity of the composites displayed percolation behavior at specific threshold values of 9.9% vol. and 4.0% vol., respectively, for EP-Cu and EP-Ni composites [75].

## 4. Conclusions

The current research endeavored to evaluate the influence of copper oxide powder, aluminum oxide powder, and graphite particles in powder and flake form on the thermal and electrical conductivity of laminate samples. Strength, fatigue, and thermal and electrical conductivity tests were conducted on laminate samples containing these admixtures, with reference material being tested without any filler. The samples were prepared manually. Density studies of the produced composites exhibited an increase in density for doped materials, with the exception of the copper powder modified laminate. The laminate with the addition of graphite in the form of powder displayed the highest density. Basic strength tests, such as a static bending test and a static tensile test, were performed on the samples. The findings of the study demonstrated that the inclusion of admixtures significantly affected the strength and stiffness of composites. Specifically, in the static bending test, the material doped with copper oxide powder exhibited the highest flexural strength, while the material modified with graphite in the form of flakes showed the highest tensile strength. Furthermore, the incorporation of graphite in this form resulted in an increase in the modulus of elasticity in both tension and bending.

High energy dissipation values in composites indicate their capacity to efficiently absorb and release energy, improving their performance in a range of applications. Numerous processes, such as matrix plasticity, interfacial slip, and component friction, all play important roles in improving the damping capacity of composite materials. These mechanisms also contribute to the dissipation of energy in composites. In the end, a high energy dissipation value in composites denotes how well they absorb and release energy, which makes them advantageous for a range of technical uses. From the research conducted, it can be concluded that the addition of graphite in the form of flakes improves the energy dissipation capacity of the composite, compared to other additives. Moreover, the impact of environmental factors does not cause the degradation of this parameter, but even has a positive effect. At this stage, it is not possible to explain this phenomenon, but it may provide a basis for future research.

The thermal and electrical conductivity tests were also carried out. The optimal outcomes were obtained following the incorporation of graphite, both in the form of powder and flakes. The highest thermal conductivity was exhibited by the material modified with graphite in the form of gunpowder, and the thermal resistance was the lowest for this additive, which facilitated thermal conduction. The findings of these investigations were corroborated by electrical conductivity tests. The addition of graphite resulted in a decrease in electrical impedance. The best results were obtained for the laminate modified with graphite in the form of gunpowder. The flake graphite also decreased the resistance to the current flowing through the sample, although the geometry and arrangement of the flakes likely impede the flow of current, causing an increase in impedance relative to the graphite-modified material in the form of gunpowder. In conclusion, a comparison of composites doped with various additives designed to enhance thermal and electrical conductivity revealed that graphite is the most effective additive. This additive will also impact the optimal ratio of thermal and electrical conductivity to mechanical strength.

## Figures and Tables

**Table 1:** Composition of manufactured materials.

Index	Description
EP	Epoxy laminate.
EP + 10% Al	Epoxy resin modified with aluminum oxide at a concentration of 10% by weight.
EP + 10% Cu	Epoxy resin modified with copper oxide at a concentration of 10% by weight.
EP + 10% Gf	Epoxy resin modified with graphite in the form of flakes at a concentration of 10% by weight.
EP + 10% Gp	Epoxy resin modified with graphite in the form of particles at a concentration of 10% by weight.

**Table 2:** Parameters of one thermal cycle.

Cycle	Function	Intensity, W/m^2^/nm	Temp., °C	Time, min
	UV light	1.55	60	8:00
	Water spraying	-	-	0:15
	Condensation	-	50	3:45

**Table 3:** Colorimetric results.

Type of Samples	ΔA After Aging Tests
EP	19.57
EP + 10% Al	0.5
EP + 10% Cu	2.59
EP + 10% Gf	2.19
EP + 10% Gp	1.49
	Legend
0 < ΔA < 1	you cannot see the difference
1 < ΔA < 2	an experienced observer sees the difference
2 < ΔA < 3.5	the inexperienced observer sees the difference
3.5 < ΔA < 5	the observer perceives a clear difference of colors
5 < ΔA	the observer perceives the colors as completely different

## Data Availability

The raw data supporting the conclusions of this article will be made available by the authors on request.
